# Enhancement of Differentiation and Mineralisation of Osteoblast-like Cells by Degenerate Electrical Waveform in an *In Vitro* Electrical Stimulation Model Compared to Capacitive Coupling

**DOI:** 10.1371/journal.pone.0072978

**Published:** 2013-09-11

**Authors:** Michelle Griffin, Anil Sebastian, James Colthurst, Ardeshir Bayat

**Affiliations:** 1 Plastic and Reconstructive Surgery Research, Manchester Institute of Biotechnology, University of Manchester, Manchester, United Kingdom; 2 Fenzian Ltd., Berkshire, United Kingdom; 3 Department of Plastic and Reconstructive Surgery, South Manchester University Hospital Foundation Trust, Wythenshawe Hospital, Manchester, United Kingdom; 4 Institute of Inflammation and Repair, University of Manchester, Manchester Academic Health Science Centre, South Manchester University Hospital Foundation Trust, Wythenshawe Hospital, Manchester, United Kingdom; Politecnico di Milano, Italy

## Abstract

Electrical stimulation (ES) is effective in enhancing bone healing, however the best electrical waveform, mode of application and mechanisms remains unclear. We recently reported the *in vitro* differential healing response of a novel electrical waveform called degenerate sine wave (DW) compared to other forms of ES. This study further explores this original observation on osteoblast cells. Here, we electrically stimulated SaOS-2 osteoblast-like cells with DW in an *in vitro* ES chamber (referred to as ‘DW stimulation’) and compared the intracellular effects to capacitive coupling (CC) stimulation. ES lasted for 4 h, followed by an incubation period of 20 h and subsequent ES for 4 additional hours. Cytotoxicity, proliferation, differentiation and mineralisation of the osteoblast-like cells were evaluated to determine the cell maturation process. DW significantly enhanced the differentiation of cells when compared to CC stimulation with increased alkaline phosphatase and collagen I gene expression by quantitative real time- polymerase chain reaction analysis (p<0.01). Moreover, DW significantly increased the mineralisation of cells compared to CC stimulation. Furthermore the transcription of osteocalcin, osteonectin, osteopontin and bone sialoprotein (p<0.05) was also up regulated by DW. However, ES did not augment the proliferation of cells. Translational analysis by immunocytochemistry and Western blotting showed increased collagen I, osteocalcin and osteonectin expression after DW than CC stimulation. In summary, we have demonstrated for the first time that DW stimulation in an *in vitro* ES chamber has a significant effect on maturation of osteoblast-like cells compared to CC stimulation of the same magnitude.

## Introduction

Since Yasuda and Fukada in 1957 demonstrated that bone displays piezoelectric properties, there has been an increasing interest in stimulating bone cell activities by exogenous electrical stimulation (ES) [1]. Despite the successful use of ES for bone healing [2]–[7] the effect of ES on osteoblast activities is still under review [8]–[16]. *In vitro* research has been focused on the effects of ES on osteoblast activity; primarily investigating the different stages of osteoblast development including proliferation, differentiation and mineralisation of osteoblasts [9], [10], [17] and how ES affects these stages [8], [9].

Interestingly, the influence of ES on proliferation of osteoblasts has been contradictory in published reports [8], [9], [18], [19]. Some studies report ES significantly enhances the proliferation of osteoblasts [8], [9], [18] whereas others have shown ES reduces osteoblast’s proliferative activity [19], [20]. The effect of ES on the differentiation of osteoblasts is also controversial with reports of decreased [9] and increased alkaline phosphatase activity after ES [8], [21], [22]. The reported effect of ES on mineralisation remains limited [10] although ES has been shown to enhance mineralisation by increasing osteoblast expression of osteocalcin, osteonectin and bone sialoprotein [21].

Electrical stimulators have been shown to enhance bone healing in many orthopaedic conditions including delayed healing or non-union fractures [4] and osteotomies [3], improving the efficacy of bone grafts [2], treating fresh fractures [7] and enhancing spinal fusion [5]. Different types of electrical stimulators vary in accordance with the applied electrical waveform, which has a characteristic frequency, amplitude and shape of the waveform. Pulsed electromagnetic field (PEMF), capacitive coupling (CC) and direct current (DC) [20] are the common modes of ES widely being used in bone healing. CC has been shown to be successful in aiding bone repair in several clinical situations including non-union [23], [24] stress fractures [25] and spinal fusion [5]. Various other types of ES [2], [4], [7] have shown to be effective in aiding bone healing for clinical practice. It is evident from reported studies that osteoblast activity depends on the specific waveform applied, albeit most studies emphasise the effect of PEMF [20].

Recently, Perry et al. [26] have established the potential role of a non-invasive ES device, called the Fenzian treatment system in the management of chronic scars, pain and itch. To further characterise the Fenzian treatment system, Sebastian et al. [27] digitised the degenerate electrical wave signal from the ES device and successfully altered the differential expression of collagen I in keloid fibroblasts in a novel i*n vitro* ES chamber, along with alternating current (AC) and DC stimulations. Additionally, Sebastian et al. [28] illustrated that cutaneous wounds receiving degenerate wave (DW) electrical stimulation display accelerated healing seen by reduced inflammation, enhanced angiogenesis and advanced remodeling phase. We have illustrated the advantage of DW application in the recruitment of bone marrow mesenchymal stem cells (BMMSCs) to the fracture site that may enhance the rate of bone healing [29]. Since the differential regulation of genes has been shown possible with DW [27], we hypothesised that DW could also influence osteoblast activities during bone healing. Therefore, this study compared the effect of DW in two different application modes, 1) an *in vitro* ES model (hereon referred to as ‘DW stimulation’) and 2) Capacitive coupling (hereon referred to as ‘CC stimulation’) on the proliferation, differentiation and mineralisation aspects of SaOS-2 osteoblast-like cells.

## Materials and Methods

### Cell culture

Human osteosarcoma cell line SaOS-2 [30] was cultured in Dulbecco's Modified Eagle's Medium (DMEM) (E15-009, PAA, Pasching, Austria) supplemented with 10% fetal calf serum (A15-152, PAA), penicillin (100 units/mL) and streptomycin (100 units/mL) (P11-010, PAA) and 2 mM L-glutamine (M11-004, PAA). Additionally, for mineralisation, cells were supplemented with 50 μg/ml ascorbic acid (A4403, Sigma, Dorset, UK) and 7.5 mM β-glycerophosphate (G9422, Sigma). Cells were incubated and grown to confluence in 0.2 µm vented T25 flasks (3289, Corning, NY, USA) at 37°C in humidified air with 5% CO_2_ before being harvested ( ∼ 7 days) with trypsin and passaged. For experiments, cells were seeded (1.5×10^5 ^cells/cm^2^) on microscopic cover slips (9 mm×9 mm) and these cover slips are referred to as ‘slides’.

### Electrical stimulation apparatus

#### (1) *In vitro* electrical stimulation apparatus

**Figure 1:**
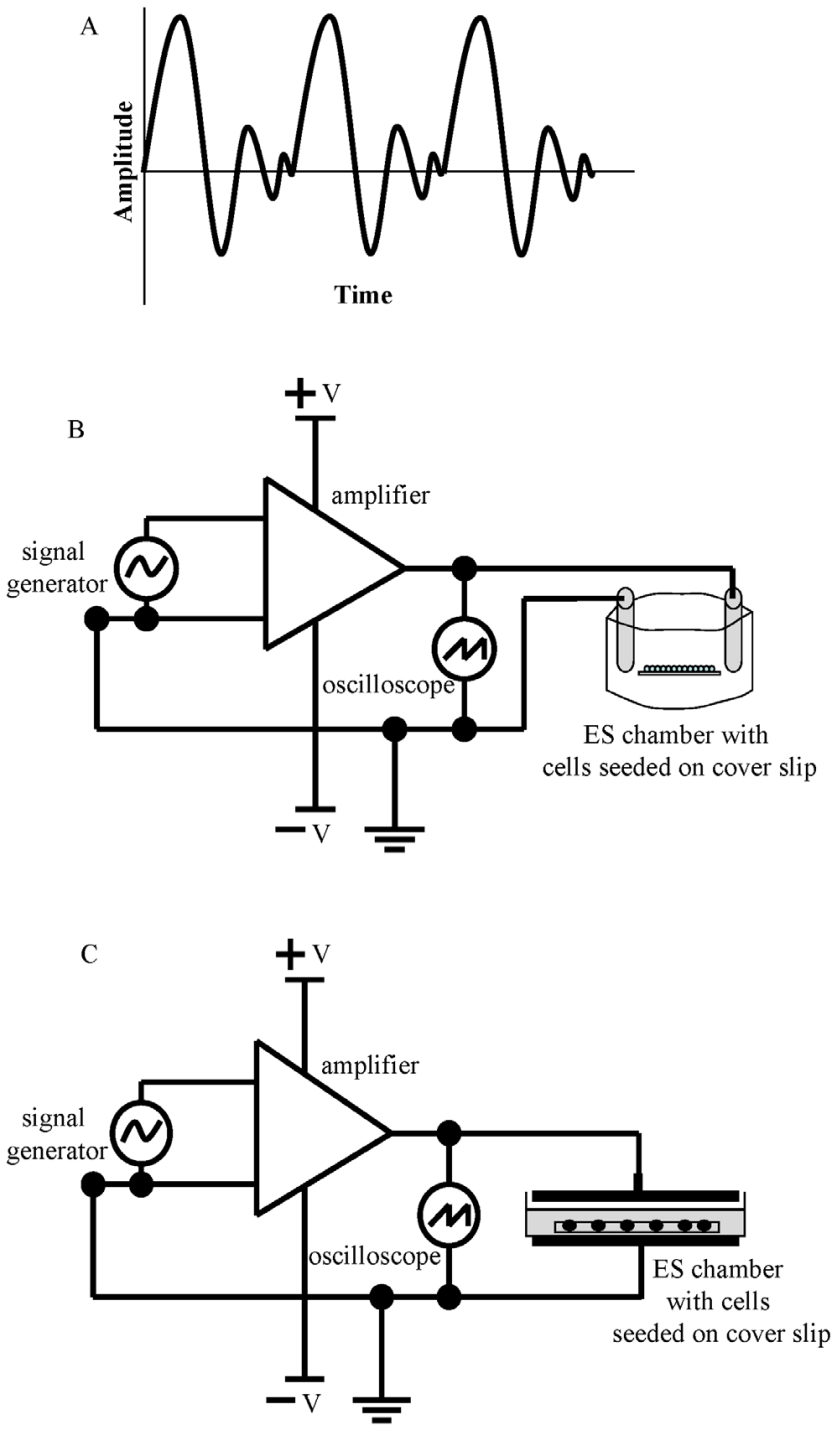
Degenerate waveform (1A) when applied to the customized *in vitro* ES apparatus with ES chamber (1B) or Capacitive Coupling stimulation apparatus (1C).

The *in vitro* ES apparatus has been used in our laboratory previously [27]. To deliver DW (Data S1 in File S2) to SaOS-2 osteoblast-like cells, an ES apparatus was designed consisting of a modified glass petri dish (rectangular glass chamber referred to as ‘ES chamber’; 44 mm×13 mm×11 mm) and the slides were placed in the ES chamber. The required electric current was conducted into the chamber through Ag–AgCl electrodes inserted in synthetic rubber – agar bridges filled with Steinberg’s saline (Data S1 in File S1) gelled with 1% agar (A/1080/48, Fisher Scientific, Loughborough, UK). Two 10-mm-diameter holes were drilled in the glass petri dish cover lid for agar bridges to enter the ES chamber. The cell monolayer was subjected to an electric field of 10 mV/mm and 16 Hz (Fig. 1A, 1B) as previously described [29]. Further description of the apparatus is provided in Data S1 in File S1.

#### (2) Capacitive coupling apparatus

For generating CC mode of degenerate wave ES, a similar system employed by Hartig et al. [21] and Wisemann et al. [10] was used. The CC electric parameters were set in such a way that the cell monolayer was subjected to an electric field of 10 mV/mm and 16 Hz (Fig. 1A, 1C; Griffin et al. [29]), similar to the *in vitro* ES chamber as explained above. Capacitors consisted of a pair of high-grade steel electrodes, each having a diameter of 8 cm, mounted in a plastic insulating material. The petri dish (with the cell seeded slides) was placed on the lower electrode and the upper electrode was placed above the medium leaving an air gap of ∼2mm. This comprises the total separation between the electrodes to ∼7.7 mm. The whole system was placed in 37°C temperature-regulated incubator box. Detailed description of the apparatus is provided in Data S2 in File S1 and electric field simulation studies using FEMLAB® 3.5 (Comsol Multiphysics Inc.) in Fig S1 (A-D).

### Protocol for electrical stimulation

After slides were placed in the *in vitro* or CC ES chamber, 2 ml of DMEM medium supplemented with 25 mM HEPES (4-(2-hydroxyethyl)-1-piperazineethanesulfonic acid) buffer (17-737E, Lonza, Slough, UK) or 20 ml DMEM medium with 25 mM HEPES was added respectively, before any initiation of ES. SaOS-2 cells were stimulated for 4 h either by CC or DW and then incubated at 37°C (5% CO_2_) for 20 h. This was mainly to observe the prolonged intracellular effects of electrical stimulation. At the 24^th^ h, ES was continued for further 4 h. At the 0, 2^nd^, 4^th^, 24^th^, 26^th^, and 28^th^ h, two slides were taken from the ES chamber and further analysed. The zero time point was before any ES was applied. Control slides were treated in the same way as the ES slides in the ES chamber, but without any stimulation.

### Cytotoxicity assay

The effect of ES on cell toxicity was determined by measuring the secretion of Lactate dehydrogenase (LDH) by damaged SaOS-2 cells using the Cytotoxicity Detection Kit (Roche Diagnostics, Burgess Hill, UK). 1.5×10^5^ cells/cm^2^ were seeded on slides and cell culture medium was collected at different time points (0, 2, 4, 24, 26 and 28 h) to quantitatively analyse the LDH activity. Control slides were treated in the same way as the ES slides in the ES chamber, but without any stimulation. At the end of the experiment, cell numbers were counted in different microscopic fields using 40X objective (0.95 mm^2^) and the values were normalised for 10^5^ cells.

### Cell proliferation assay

The effect of ES on cell proliferation was determined by measuring the secretion of 2-(4-Iodophenyl)-3-(4-nitrophenyl)-5-(2,4-disulfophenyl)-2H-tetrazolium salt (WST-1) by SaOS-2 cells using the cell proliferation detection kit (Roche Diagnostics). 1.5×10^5 ^cells/cm^2^ were seeded on slides and reconstituted WST-1 mixture was added to the slides at different time points (0, 2, 4, 24, 26 and 28 h) to quantitatively analyse the WST-1 activity. Control slides were treated in the same way as the ES slides in the ES chamber, but without any stimulation. At the end of the experiment, cell numbers were counted in different microscopic fields using 40X objective (0.95 mm^2^) and the values were normalised for 10^5^ cells.

### Alkaline phosphatase (ALP) assay

To determine the effect of ES on the ALP activity of the SaOS-2 cells the ALP reagent kit was used (DALP-250, Bioassay systems, Hayward, CA, USA). 1.5×10^5^ cells/cm^2^ were seeded on slides. The level of ALP activity was evaluated by analysing 500 µl of cell growth medium at each time point (0, 2, 4, 24, 26 and 28 h). For measuring intracellular ALP, the cell grown slides were washed with PBS, after which the cells were lysed in 0.5 mL 0.2% Titron X-100 (HFH-10, Invitrogen, Paisley, UK). The cell lysate was shaken for 20 min at room temperature. Subsequently, 150 µl of cell lysate or cell medium was transferred to a 96-well plate, which was supplemented with 50 µl of p-nitrophenol phosphate (p-NP) substrate. The formation of p-NP was quantified by measuring the optical density using a microplate reader at a wavelength of 405 nm. Before lysing cells to measure intracellular ALP, cell numbers were counted in different microscopic fields using 40X objective (0.95 mm^2^) and the values were normalised for 10^5^ cells.

### Alizarin Red S (ARS) staining and Cetylpyridinium chloride (CPC) extraction

Mineralisation was analysed by quantifying the formation of calcium phosphate by SaOS-2 cells (0, 2, 4, 24, 26 and 28 h) using ARS staining based on the protocol described by Jensh et al. [31] and Martino et al. [30]. 1.5×10^5 ^cells/cm^2^ were seeded on slides. Briefly, cells were washed with Dulbecco’s PBS and fixed with 100% methanol for 10 min at –20°C. Afterwards cells were washed and stained with 40 mM ARS solution (A5533, Sigma) for 10 min and subsequently washed with sodium acetate buffer solution (pH 6.3). Washing with PBS was continued till the cells were all clear off the stain debris. For quantifying ARS retained in the cells, the cell-grown slides were incubated at room temperature with 500 µl of 10% (w/v) CPC (C0732, Fisher Scientific) for 1 h. The dye solution was transferred to a 96-well plate to measure the absorbance at 570 nm. At the end of the experiment, cell numbers were counted in different microscopic fields using 40X objective (0.95 mm^2^) and the values were normalised for 10^5^ cells.

### Von Kossa staining

The control and 28 h cells were washed with PBS, fixed in phosphate buffered formalin for 10 min and washed with water. They were serially dehydrated in 70%, 95% and 100% ethanol and air dried. The cell cultures were rehydrated by 100%, 95% and 80% ethanol and finally in water. The water was removed, 2% silver nitrate solution (S6506, Sigma) added and the cell cultures were exposed to sunlight for 20 minutes and the plate was rinsed with water. 5% sodium thiosulphate (217263, Sigma) was added for 3 minutes, rinsed in water and counter stained with Van Geisan (Picrofuchsin, HX071948, Merck) for 5 minutes. The cells were washed with water, 95% and 100% ethanol and dried for image analysis [32]. At the end of the experiment, nodular staining was counted in different microscopic fields using 40X objective (0.95 mm^2^).

### Quantitative Real Time-Polymerase Chain Reaction

Cells were extracted from slides using TRIzol Reagent (155960, Invitrogen). The RNeasy kit (74106, Qiagen, West Sussex, UK) was used to extract cellular RNA and NanoDrop ND-1000 UV-visible spectrophotometer (Labtech International, East Sussex, UK) was used to estimate the total RNA concentration. RNA was normalised for all the different samples to 500 ng for cDNA synthesis. Transcriptor first strand cDNA synthesis kit (Roche applied science) was used for cDNA synthesis and cDNA was quantified again with Nanodrop machine (Labtech International, East Sussex). Quantitative real-time polymerase chain reaction (qRT-PCR) was performed as described previously [27]. qRT-PCR was carried out using the LightCycler 480 II platform (Roche Diagnostics, Germany). Each qRT-PCR reaction was carried out in a final volume of 10 µl, consisting of 4 µl (5 ng cDNA) diluted template cDNA, 5 µl Light Cycler 480 probes master mix (Roche Diagnostics), 0.2 µM of forward and reverse primers (Sigma; Table S1 in File S1), 0.1 µl probe from Universal Probe Library (Roche Diagnostics) and 0.5 µl nuclease-free water (AM9922, Ambion, life technologies, Paisley, UK). White 96-well plates (Roche Diagnostics) were used for all the experiments and each reaction was done in triplicate. The reactions were initiated at 95°C for 10 minutes. Each of the 40 amplification cycles consisted of a 10 s denaturation step at 95°C and a 30 s annealing and elongation step at 60°C. The fluorescence intensity was recorded at the end of the annealing step and elongation step in each cycle. After the 40 cycles of amplification, a cooling step at 40°C for 30 s was carried out. The gene expression levels were normalised with an internal reference gene, ribosomal protein L32 (RPL32). Relative gene expression was calculated with the formula, G_r_  =  2?(–ΔC^T^). Here, ΔC^T^ is the difference in threshold cycle values between the target gene and RPL32.

### Immunocytochemistry (ICC)

Fluorescence ICC was performed for osteoclacin and collagen I, and peroxidase ICC was performed for ALP using the R.T.U Vectastain kit (PK-7200, Vector laboratories, Burlingame, CA, USA). The primary antibodies, secondary antibodies, concentration of antibodies, incubation time and detection methods are detailed in Table S2 in File S1. For immunoperoxidase experiments, nuclei counterstaining was done using Mayer’s haematoxylin (01560BBE, Surgipath Europe, Peterborough, UK) and the slides were finally mounted with Shandon Consul-Mount (99-904-40, Thermo Scientific, Hemel Hempstead, UK).

### Western blotting (WB)

Total cell lysate (60 micrograms) from osteoblast-like cells (control 28 h, CC 28 h and DW 28 h) were separated using 12% sodium dodecyl sulphate- polyacrylamide gel electrophoresis and transferred to polyvinylidene difluoride membrane using iBLOT apparatus (IB4010-01, Invitrogen). The primary antibodies, secondary antibodies, concentration of antibodies, incubation time and detection methods are detailed in Table S3 in File S1.

### Statistical methods

Data is presented as mean +/– standard deviation from three independent experiments performed in triplicates (n = 3). Statistical analysis was calculated using one way ANOVA for comparison between three groups with Turkey post hoc test and student t test for comparison between two groups. Confidence intervals of 95% with corresponding *p* value of 0.05 was chosen throughout analysis. * p<0.05; ** p<0.01; *** p<0.001; $ p<0.0001; £ p > 0.05.

## Results

### CC has an increased cytotoxic and decreased proliferative effect on SaOS-2 osteoblast-like cells

**Figure 2:**
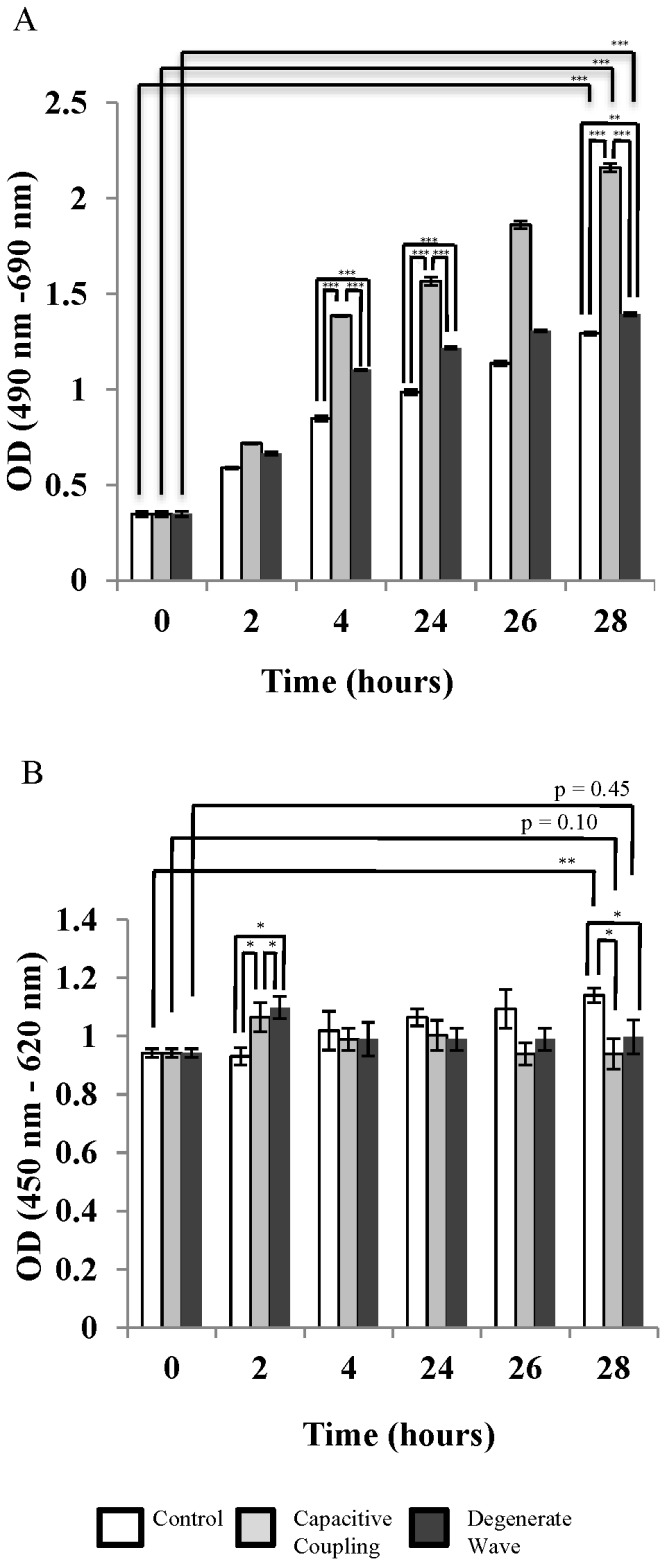
Cytotoxicity by LDH assay (2A). The cytotoxicity was greater for the osteoblast-like cells exposed to CC stimulation compared to DW stimulation. Cell proliferation by WST-1 assay (2B). Cell proliferation was greater for non-stimulated cells compared to cells exposed to electrical stimulation by 28 h. * p <0.05; ** p <0.01; *** p <0.001. OD  =  optical density.

There was an increase in cytotoxicity after 28 h for SaOS-2 cells with and without ES as shown by LDH (Fig. 2A). By the 28^th^ h, the cytotoxicity was significantly higher by CC stimulation compared to DW (p< 0.0001) and the non-stimulated cells (p<0.0001). After two h, the proliferation was significantly increased for cells with ES compared to the non-stimulated cells, with DW significantly (p< 0.001) enhancing the proliferation compared to CC and the non-stimulated cells (Fig. 2B). However, proliferation of cells without ES was significantly higher (p<0.001) in comparison to ES cells at the end of the 28^th^ h. This demonstrates the transient effect of ES in inducing cell proliferation on osteoblast-like cells.

### DW increased the differentiation of SaOS-2 osteoblast-like cells compared to CC

**Figure 3:**
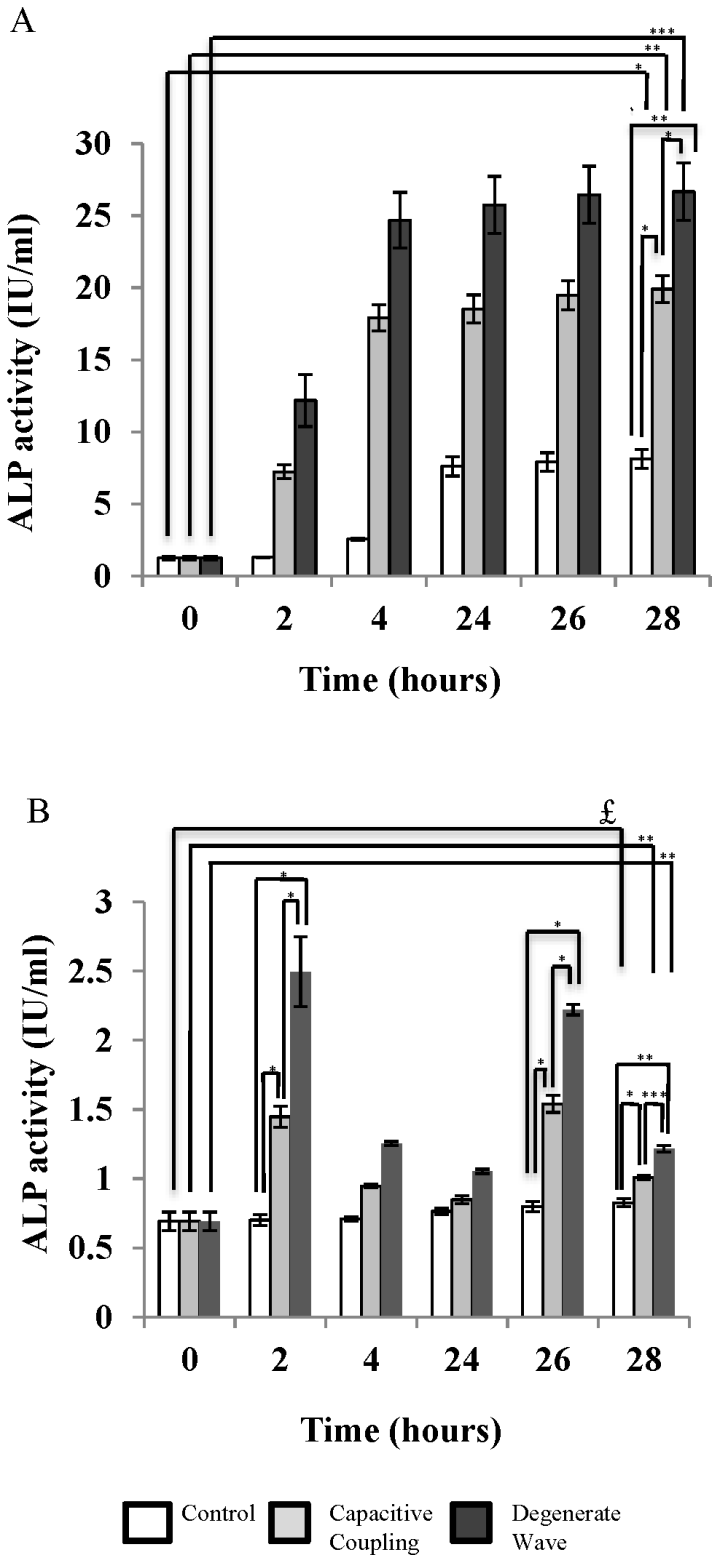
Alkaline phosphatase (ALP) activity in the cell growth medium (3A). ALP activity was greater after DW stimulation compared to CC stimulation by 28 h. Intracellular ALP activity (3B). ALP activity was greater after DW stimulation compared to CC stimulation. * p <0.05; ** p <0.01; *** p <0.001.

ALP activity in the cell growth medium was significantly higher (p <0.001) for cells with ES compared to non-stimulated cells. However, DW showed higher ALP activity in cell growth medium compared to CC (Fig. 3A). At the 28^th^ h, the ALP activity in the cell growth medium had significantly increased for cells exposed to DW compared to cells exposed to CC (p<0.001) and non-stimulated cells (p<0.001; Fig. 3A). Furthermore, intracellular ALP activity was significantly higher for cells exposed to DW compared to CC and non-stimulated cells (DW vs. control p<0.0001, DW vs. CC p<0.0001) at 28 h (Fig. 3B).

### Enhanced mineralisation effect in SaOS-2 osteoblast-like cells exposed to DW compared to CC

**Figure 4:**
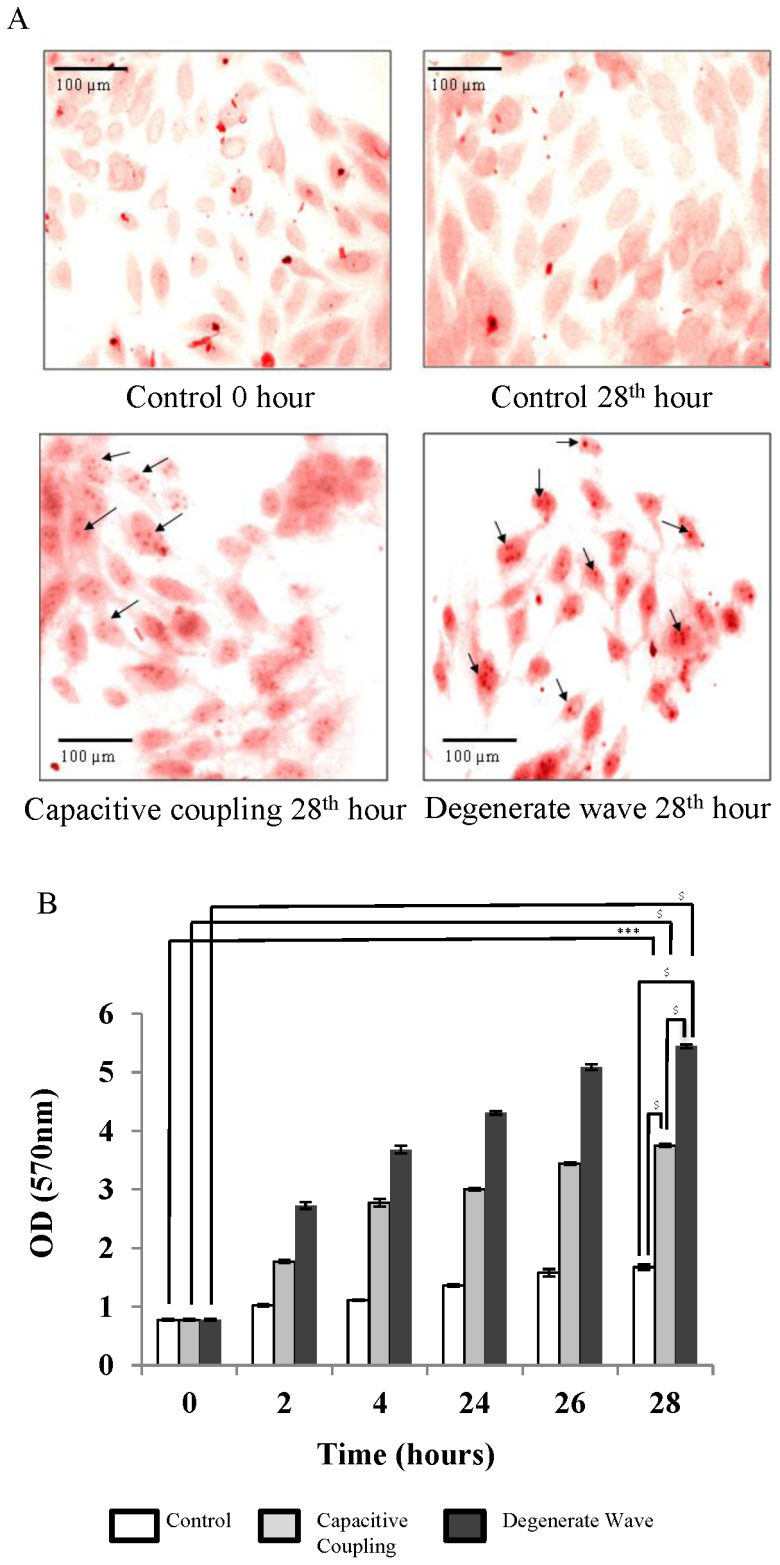
Mineral nodule formation (400x magnification) after Alizarin Red S (ARS) staining (4A). Few mineral nodules could be observed for cells without ES. A greater number of mineral nodules were observed after DW and CC by 28 h with more intense staining for cells exposed to DW than CC. Semi-quantitative analysis of intracellular ARS by CPC extraction method (4B). ARS staining was greater for cells exposed to DW than CC stimulation by 28 h. *** p <0.001; $ p<0.0001.

Cells were stained with ARS to analyse the effects of ES on osteoblast-like cell mineralisation (Fig. 4A). For the non-stimulated cells, mineral nodules were not observed even after 28 h. When cells were exposed to DW and CC stimulation, a few small nodules could be observed after 4 h, which increased in number and size by the 28^th^ h. An average of 2–3 nodules per cell, 1–2 µm in diameter were observed after both DW and CC stimulation by 28 h. Semi-quantitative analysis of the ARS stained calcium nodules indicated an increased number of calcium nodules in DW stimulated cells compared with cells exposed to CC. CPC extraction of ARS stain from the cells confirmed that mineralisation was higher in ES cells compared with the non-stimulated cells (p<0.001; Fig. 4B). Mineralisation had significantly increased at the 28^th^ h for the ES cells, with a 7-fold increase for cells exposed to DW (p<0.0001) and a 4.8-fold increase for the cells exposed to CC (p<0.001) compared to non-stimulated cells.

**Figure 5:**
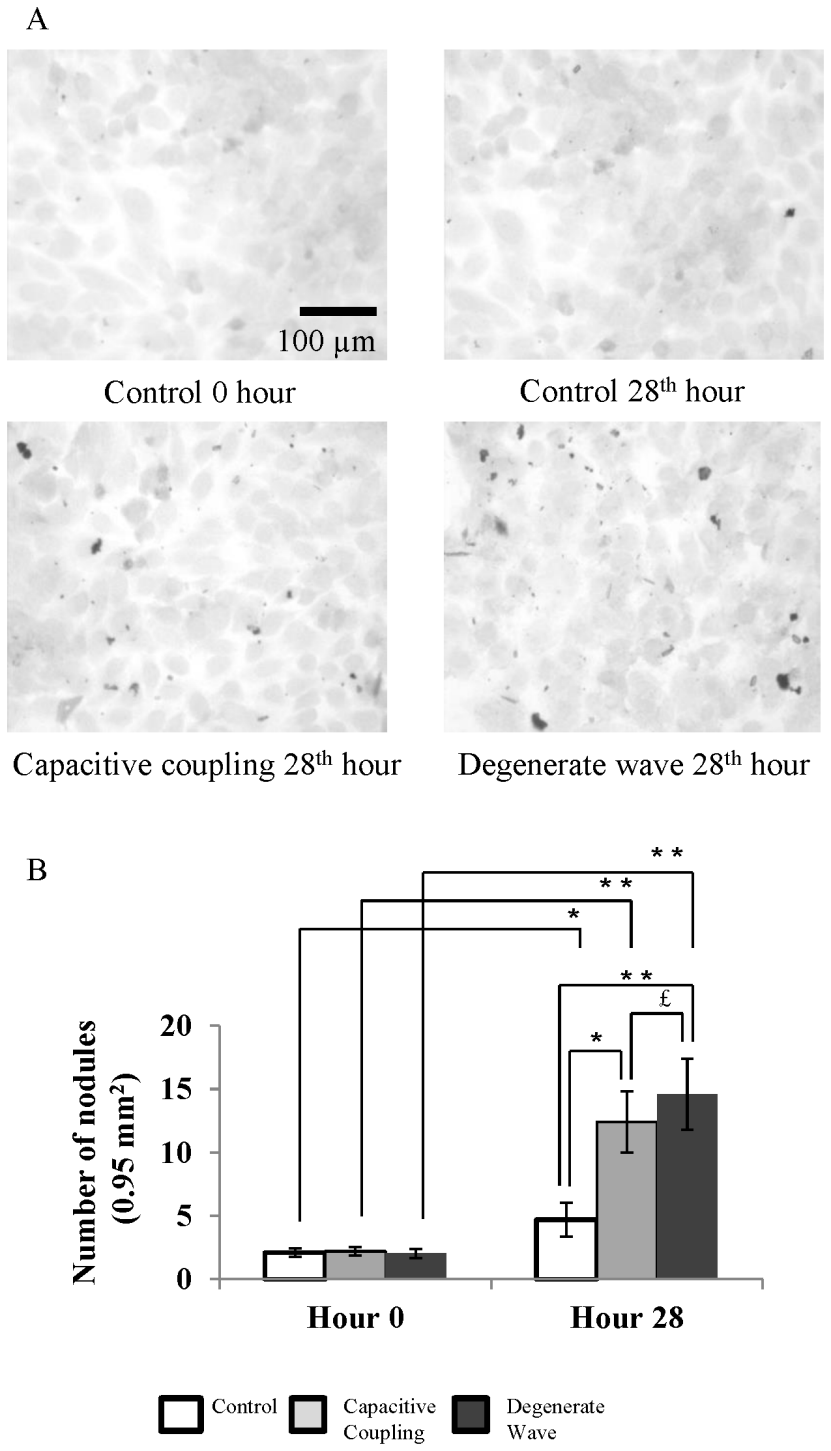
Mineral nodule formation (400x magnification) after von Kossa staining (5A). Mineral nodules were increased post ES compared to control samples. Higher number of nodules was present after DW stimulation compared to CC stimulation (5B).

Similarly, cells were also stained with von Kossa to understand mineralisation effects post ES (Fig. 5A and 5B). Albeit there was an increase in nodule formation for ES samples compared to control samples on the 28^th^ h, there was no significant difference between the two ES modes.

### qRT-PCR, Immunocytochemistry and Western blotting results demonstrate ES has a greater effect on the differentiation and mineralisation of osteoblast-like cells

**Figure 6:**
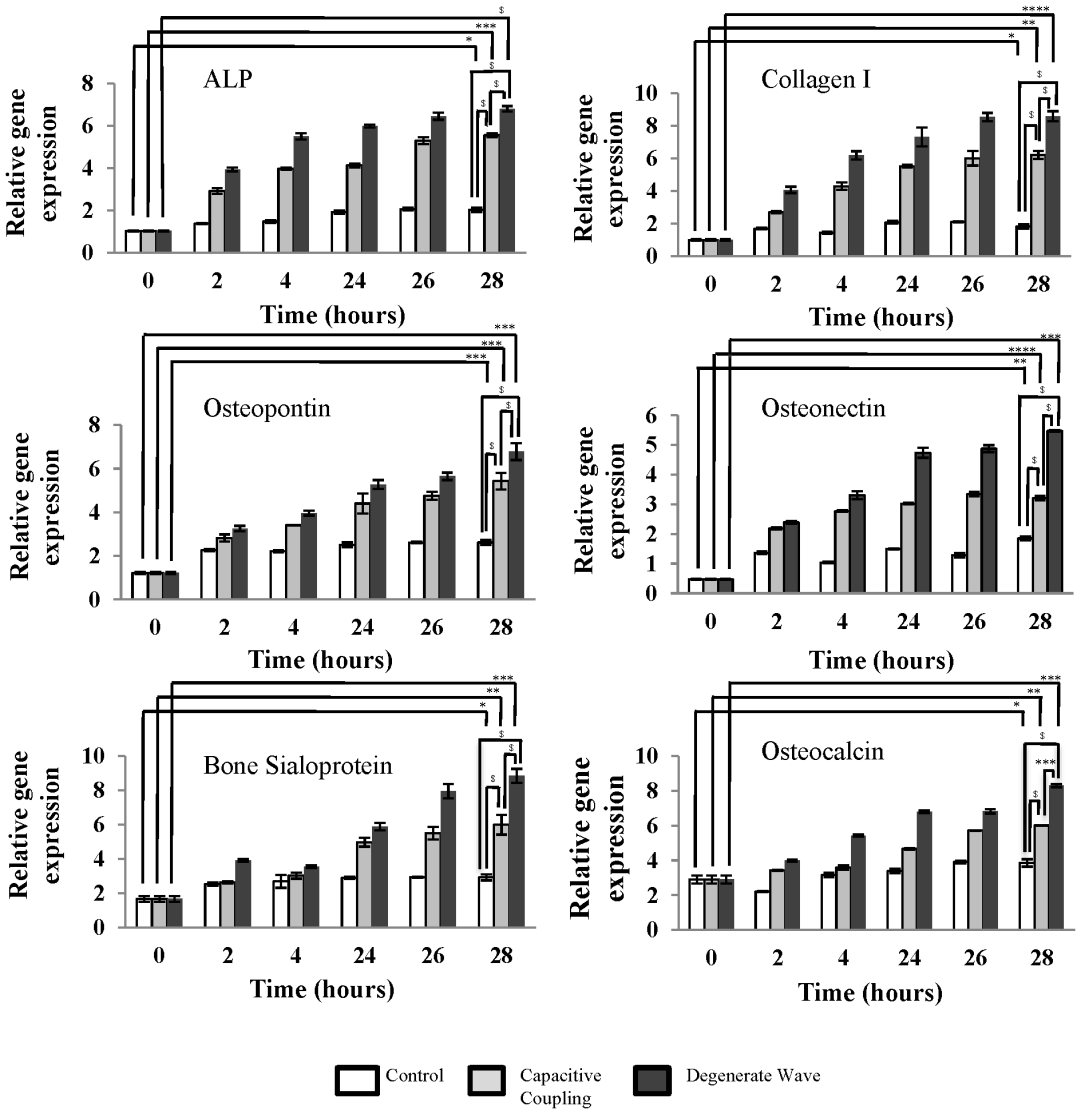
qRT-PCR analysis of differentiation and mineralisation-specific biomarkers in osteoblasts. Expression of differentiation-specific genes such as ALP, Collagen I and mineralisation-specific genes such as osteopontin, osteonectin, bone sialoprotein and osteocalcin reveal that differentiation and mineralisation of osteoblasts was greater after DW stimulation than CC stimulation by 28 h. Expression of mineralisation-specific genes by the osteoblast-like cells was greater after DW stimulation than CC stimulation by 28 h. * p <0.05; ** p <0.01; *** p <0.001; $ p<0.0001.

**Figure 7:**
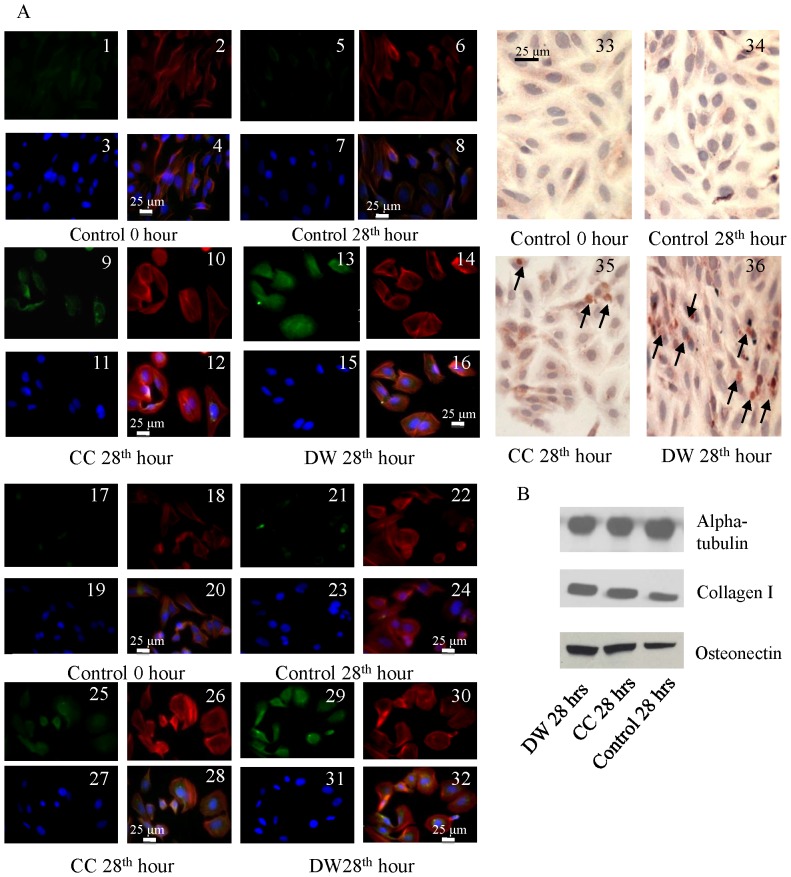
Differentiation studies of collagen I expression by ICC after 28 h of stimulation (A; 1-16). Green fluorescence is collagen I, red fluorescence is F-actin staining by rhodamine-phalloidin and blue fluorescence is DAPI staining of the nucleus. Mineralisation studies of osteocalcin expression by ICC after 28 h of stimulation (A; 17-32). Green fluorescence is osteocalcin, red fluorescence is F-actin staining by rhodamine-phalloidin and blue fluorescence is DAPI staining of the nucleus. Differentiation analysis from ALP expression by immunoperoxidase staining method after 28 h of stimulation (A; 33–36). Western blot analysis for differentiation (collagen I) and mineralisation (osteonectin) after 28 h of stimulation (B). The scale bars (25 µm) are same for the different image panels.

By 28 h, the gene expression of collagen I and ALP biomarkers were significantly higher for osteoblast-like cells exposed to DW compared to CC (p<0.0001) and non-stimulated cells (p<0.0001; Fig. 6). Moreover significant over expression (p<0.01) in the mineralisation-specific genes such as bone sialoprotein, osteopontin, osteonectin and osteocalcin were observed in DW stimulated and non-stimulated samples in comparison to CC stimulated samples (Fig. 6). From immunocytochemical (ICC) assays, collagen I, ALP and osteocalcin were over expressed by SaOS-2 osteoblast-like cells after DW electrical stimulation showing increased differentiation and mineralisation effects compared to CC (Fig. 7A). Western Blotting analysis with osteonectin and collagen I on SaOS-2 osteoblast-like cells showed over expression of these markers in ES cells compared to non-stimulated cells (Fig. 7B)**.**

## Discussion

The primary aim of this paper was to identify the effect of electrical stimulation on specific osteoblast activities, which complement events during bone healing. This is the first study to compare the effect of degenerate waveform in two different application modes, on different osteoblast activities including proliferation, differentiation and mineralisation. To date, recent studies have focused on the use of direct current and PEMF [11]–[13] with few studies reporting the effect of CC on osteoblast function [10], [21]. This novel study has demonstrated through the differential expression of genes and proteins that DW electrical stimulation in the *in vitro* ES chamber (to the same magnitude as CC stimulation), can further accelerate the maturation stages [17] of osteoblast-like cells compared to CC stimulation.

ALP is considered as an early biomarker of osteoblast differentiation [30] and vital in assembling the matrix competent for mineralisation by increasing the local phosphate concentration required for the initiation of hydroxyapatite formation [9]. From our studies, DW increased ALP activity to a greater extent than CC as demonstrated by the ALP assay. The intracellular expression level of ALP for cells exposed to ES decreased from the 2^nd^ till the 24^th^ h and from the 26^th^ to the 28^th^ h. This decline in ALP activity could be partly attributed to the biphasic response of ALP with higher expression during early osteoblast differentiation and lower response during late differentiation when mineralisation initiates. The increase in ALP from 0 to 2^nd^ h, and from 24^th^ to 26^th^ h seems to be general acceleration effect of ES on differentiation process with minimal effect on proliferation. There was a continuous rise in ALP activity in the cell growth medium for ES cells and non-stimulated cells. The overall increase in ALP post ES is in agreement with previous studies [11], [12], [19], [33], [34]. Quantitative analysis of intracellular ALP activity in SaOS-2 cells by Martino et al. demonstrates its increase by 43% compared to non-stimulated cells post PEMF application extending 4 h per day [30]. Intracellular ALP production has also shown to significantly increase after PEMF stimulation for 7 days in human osteoblast cultures [12] and to 1.7 fold after 21 days in osteoblast cells using direct current at 200 μA for 4 h per day [13]. Therefore, the optimal timing and mode of stimulation to enhance ALP activity *in vitro* in osteoblasts varies with specific modes and protocols for electrical stimulation.

Collagen I comprises 90% of the bone matrix and is expressed during differentiation of osteoblasts [17]. Our qRT-PCR analysis verified that collagen I gene expression is significantly up regulated by cells exposed to DW and CC demonstrating that ES can enhance differentiation of osteoblast-like cells (p <0.0001; Fig. 6). The effect of CC on collagen I synthesis is supported by Hartig et al. [21] where CC stimulation for 24 h increased collagen I levels in bovine osteoblast cells. Moreover, PEMF and sinusoidal electromagnetic field stimulation [35] on MG63 osteoblast cell line also had a similar effect on collagen I production [19].

Mineralisation is considered to mark the end of the osteoblast differentiation [17], where specific genes such as bone sialoprotein, osteonectin, osteocalcin, osteopontin are induced [36]. The expression of these mineralisation-specific genes by osteoblasts was significantly increased as mineral nodule formation was enhanced. These genes are responsible for regulating mineralisation thereby confirming that ES accelerates the biomineralisation process and enhances the maturation of the osteoblast [36]. From transcriptional and translational analysis, we observed that DW increases mineralisation to a higher extent than CC. ARS and von Kossa stain primarily detect calcium/hydroxyapatite crystals and further analysis on phosphates was not performed in this study. Protein analysis of osteonectin and osteocalcin indicated concomitant increase in mineralisation supported with ARS/von Kossa staining results. ES by PEMF has similarly been reported to increase the expression of osteopontin and osteocalcin expression in C3H10T1/2 osteoblast cell line after continuous exposure at a frequency of 27.1 MHz for 7 days [11] and osteocalcin after 8 h in the osteoblast cell line MG63 [20]. Therefore, various ES protocols have increasingly been reported to enhance mineralisation of osteoblasts.

In our studies, ES increased mineral nodule formation after 4 h in SaOS-2 cells in a similar pattern compared to that observed in calf osteoblast cells after 24 h of asymmetric saw-tooth voltage delivered CC stimulation [10]. Similarly, it has been reported that mineralisation increases by a magnitude of 2.5 times after 8 h of PEMF stimulation on SaOS-2 cells [30]. However, mineralisation was not observed until the second day by PEMF stimulation [30] unlike the presence of mineral granules, which were detected within 4 h of electrical stimulation in our study. Furthermore, PEMF was shown to enhance the number (39% greater when compared to non-stimulated cells) and size (70% larger compared to non-stimulated cells) of mineralized nodules of osteoblast cells compared to non-stimulated cells [30].

The observed effect of DW and CC on SaOS-2 osteoblast-like cell activity is shown in Table 1. We observed enhancement in differentiation and mineralisation of the cells, but with a limited effect on proliferation. After 4 h of ES, we observed a transient increase in proliferation compared to control cells. This could be a cell-type dependent effect as we observed contradictory results with BMMSCs [29]. Previous studies have not reported any effect on proliferation of the osteoblasts by ES [8], [30]. For osteoblasts to be able to proceed through their maturation stages of differentiation, proliferation has to be down regulated as previously described by Stein et al. [17]. Out of the three principal periods of osteoblast developmental sequence such as proliferation, matrix development and maturation, and mineralisation, the latter two stages involve differentiation and maturation. Higher expression of differentiation related genes implies the transition to later stages by down regulating the expression of genes in the initial stages in the developmental sequence of the osteoblast cell [17]. Moreover, in our study, proliferation of the cells without ES was observed to be higher than cells receiving ES. This effect has been similarly reported for osteoblast cells exposed to PEMF, where cells exhibited reduced proliferation but an enhanced differentiation phenotype [11], [19], [33], [34]. This implies the inclination of osteoblasts towards the maturation stages post ES.

**Table 1:** The effect of DW and CC stimulations on SAOS-2 osteoblast-like cells.

Electrical stimulation	Effect on SaOS-2 cells*		
	Proliferation	Differentiation	Mineralisation
**DW stimulation**	5–15% down regulated	40% up regulated	200% up regulated
**CC stimulation**	10–20% down regulated	20% up regulated	100% up regulated

* All values are in comparison to non-stimulated SaOS-2 cells.

Both waveforms decreased the proliferation of the osteoblast-like cells. However, DW enhanced the mineralisation and differentiation to a greater effect than CC which could accelerate the cell maturation process to a greater effect.

As discussed, various modes of ES trigger the multistep process of osteoblast maturation with the underlying mechanism at the cellular and subcellular levels being unknown. Moreover, it is vital to characterise various exogenous ES parameters that counteract cells to induce cytobiological reactions. In our present study, the multi-domain current interaction in CC stimulation was delineated through Femlab simulation and the single domain current path in DW stimulation was already characterised [27]. The electrodes that deliver the waveform are in contact with the cells in DW mode compared to an intermittent air interface in CC mode. Therefore location of the electrode or current source and current characteristics could be of clinical interest in electrical stimulation which potentially alters the fate of cells, accelerating and signalling the maturation stages of osteoblasts and finally exhibiting a different phenotype. Even a slight alteration in the external electric field has significant influence on the lateral electrophoretic movement and redistribution of proteins within the cell plasma membrane [37]. This prior mechanism is subsequently followed by cytoskeletal rearrangement [38], redistribution of integrins, membrane polarization and protrusions [39], changes in Ca^2+^ fluxes [40], and a cascade of adhesion mechanisms including activation of protein kinases [38] which ultimately determines cell fate. The biomarkers involved during the course of this alteration could be numerous, a few of which have been investigated in this study. The results from our *in vitro* study needs further validation in primary osteoblasts and animal models for DW to be categorised as a safe, controlled and effective application for bone healing. Future confirmational studies in the existing *in vitro* models can include knocking down relevant genes, which have been shown to have a significant effect in signalling pathways, enabling further elucidation of osteoblast proliferation and differentiation.

## Supporting Information

Figure S1 (DOCX)

File S1 Supplementary Tables.
(DOCX)

File S2 Data S1 and Data S2.
(DOCX)
