# Expression of Concern: Enhancement of Differentiation and Mineralisation of Osteoblast-like Cells by Degenerate Electrical Waveform in an *In Vitro* Electrical Stimulation Model Compared to Capacitive Coupling

**DOI:** 10.1371/journal.pone.0341207

**Published:** 2026-01-20

**Authors:** The PLOS One Editors

After this article [1] was published, concerns were raised that the Fig 5A Control 0 hour panel appears to partially overlap with the Fig 5A Control 28^th^ hour panel. Authors MG and AB stated that the Fig 5A Control 0 hour panel is incorrect and provided an updated version of Fig 5A in which the correct Control 0 hour panel is shown (S1 File). The original image data underlying Fig 5A are presented in the S2 File.

During post-publication discussions, authors MG and AB provided quantitative data underlying the results in [1]. Upon editorial review, PLOS identified additional concerns with these data as well as with the statistical analysis reported in this article. PLOS will be investigating the concerns for the underlying data and statistical analyses in accordance with COPE guidance and journal policies. Meanwhile, the *PLOS One* Editors issue this Expression of Concern.

## Supporting information

S1 File Replacement Fig 5A. (TIF)

S2 File Image data underlying Fig 5A. (ZIP)
