# Supplementary material for: Enhancement of Differentiation and Mineralisation of Osteoblast-like Cells by Degenerate Electrical Waveform in an In Vitro Electrical Stimulation Model Compared to Capacitive Coupling
Source: PLoS One. 2013 Sep 11;8(9):e72978. doi: 10.1371/journal.pone.0072978 (PMC3770651; doi:10.1371/journal.pone.0072978)
Supplement: Figure S1 — (DOCX) [file pone.0072978.s001.docx]

**Supplementary Figure A: General Femlab simulation.** Femlab electric potential simulation for capacitive coupling mode with a single cell seeded on a glass cover slip.

**Supplementary Figure B: Femlab simulation with assigned domains.** Femlab electric potential simulation for capacitive coupling mode with assigned domains.

**Supplementary Figure C: Femlab simulation on a localised single cell**. Localised image of a mammalian cell (10 µm thickness and 100 µm length) seeded on top of a glass cover slip, simulated for electric potential using Femlab.

**Supplementary Figure D: Electric potential simulation of single cell in capacitive coupling mode.** We obtained a value of nearly 103 µV on the single cell when the capacitors were assigned 160 mV.
