# Supplementary material for: Enhancement of Differentiation and Mineralisation of Osteoblast-like Cells by Degenerate Electrical Waveform in an In Vitro Electrical Stimulation Model Compared to Capacitive Coupling
Source: PLoS One. 2013 Sep 11;8(9):e72978. doi: 10.1371/journal.pone.0072978 (PMC3770651; doi:10.1371/journal.pone.0072978)
Supplement: File S1 — Supplementary Tables. (DOCX) [file pone.0072978.s002.docx]

| **Gene** | **Primers (bp)**  **FP: Forward primer**  **RP: Reverse primer** | **Accession number** | **Product Size (bp)** |
| --- | --- | --- | --- |
| Bone Sialoprotein (BSP) | FP: caatctgtgccactcactgc (20)  RP: tcattttggtgattgcttcct (21) | NM_004967.3 | 74 |
| Osteonectin (ON) | FP: gtgcagaggaaaccgaagag (20)  RP: tgtttgcagtggtggttctg (20) | NM_003118.2 | 64 |
| Osteocalcin (OC) | FP: tgagagccctcacactcctc (20)  RP: acctttgctggactctgcac (20) | NM_199173.3 | 98 |
| Osteopontin (OPN) | FP: cgcagacctgacatccagt (19)  RP: ggctgtcccaatcagaagg (19) | NM_001040058.1 | 127 |
| Collagen type 1 (Col 1) | FP: gggattccctggacctaaag (20)  RP: ggaacacctcgctctcca (18) | NM_000088.3 | 63 |
| Alkaline Phosphatase (ALP) | FP: cgttggtgttgagcttctga (20)  RP: cctgccttactaactccttagtgc (24) | NM_000478.3 | 112 |
| RPL32 | FP: gaagttcctggtccacaacg (20)  RP: gagcgatctcggcacagta (19) | NM_000994.3 | 77 |

**Supplementary Table S1: Details of primers used for qRT-PCR.**

| **Primary antibody name** | **Raised species** | **Isotype** | **Dilution** | **Product code** | **Company** | **Primary antibody incubation** | **Secondary antibody name**  **(concentration)**  **(Incubation)** | **Detection method** |
| --- | --- | --- | --- | --- | --- | --- | --- | --- |
| Type 1 Collagen | Rabbit (polyclonal) | IgG | 1:300 | ab59435 | Abcam, Cambridge, UK | 4^o^C overnight | Anti- Rabbit Alexa 488  (1:500)  (1h room temp) | fluorescence |
| Osteocalcin | Mouse (monoclonal) | IgG2 | 1:200 | ab13419 | Abcam, UK | 4^o^C overnight | Goat anti-mouse Cy3 conjugated (1:200)  (1h room temp) | fluorescence |
| Alkaline Phosphatase | Rabbit  (polyclonal) | IgG | 1:100 | ab75699 | Abcam, UK | 4^o^C overnight | Universal secondary antibdy | peroxidase |

**Supplementary Table S2:** **Antibody information for immunocytochemistry**. Primary antibodies, secondary antibodies, concentration of antibodies, incubation time and detection methods used for immunocytochemistry analysis.

| **Primary antibody name** | **Raised species** | **Isotype** | **Dilution** | **Product code** | **Company** | | **Primary antibody incubation** | **Secondary antibody name**  **(concentration)**  **(Incubation)** | **Detection method** |
| --- | --- | --- | --- | --- | --- | --- | --- | --- | --- |
| Osteonectin | Mouse (monoclonal) | IgG1 | 1:500 | ab89218 | Abcam, UK | 4^o^C overnight | | anti-mouse IgG  (1:1000)  (1h room temp) | chemiluminescence ECL kit  (RPN2132, GE Healthcare, Buckinghamshire, UK) |
| Type 1 Collagen | Rabbit (polyclonal) | IgG | 1:500 | ab59435 | Abcam, UK | 4^o^C overnight | | anti-rabbit IgG  (1:1000)  (1h room temp) | chemiluminescence ECL kit |
| Alpha tubulin | Mouse (monoclonal) | IgG1 | 1:500 | ab7291 | Abcam, UK | 4^o^C overnight | | anti-mouse IgG  (1:1000)  (1h room temp) | chemiluminescence ECL kit |

**Supplementary Table S3:** **Antibody information for Western blotting**. Primary antibodies, secondary antibodies, concentration of antibodies, incubation time and detection methods used for Western blotting.
