# Supplementary material for: Enhancement of Differentiation and Mineralisation of Osteoblast-like Cells by Degenerate Electrical Waveform in an In Vitro Electrical Stimulation Model Compared to Capacitive Coupling
Source: PLoS One. 2013 Sep 11;8(9):e72978. doi: 10.1371/journal.pone.0072978 (PMC3770651; doi:10.1371/journal.pone.0072978)
Supplement: File S2 — Data S1 and Data S2. (DOCX) [file pone.0072978.s003.docx]

**Supplementary Data S1**

***In vitro* electrical stimulation apparatus (Sebastian et al. [2011])**

Steinberg’s saline is 60 mM NaCl, 0.7 mM KCl, 0.8 mM MgSO_4_.7H_2_O, 0.3 mM CaNO_3_.4H_2_O, 1.4 mM Tris base maintained at pH 7.4. A pair of made-up Ag-AgCl electrodes were introduced inside the agar bridges in order for the required current to be achieved without excessive power dissipation (this system is not cytotoxic). This end of the electrode was wrapped with agar in translucent silicone tubing (customised to 2 cm length, outer diameter 10 mm, Fisher Scientific, Loughborough, UK) and introduced into the electrical stimulation (ES) chamber. The agar electrodes were in close proximity to the cell grown slides (11 mm x 25 mm). 2.5 ml of cell growth medium was introduced into the chamber. The other end of the silver electrode was connected to the power supply. The degenerate wave (DW) signals were programmed and copied on the signal generator (Agilent waveform generator; 33220A, Agilent technologies, Wokingham, UK) which produced the required waveform. The amplifier used was Prism LA150M (Otley, West Yorkshire, UK).

**Degenerate waveform (Sebastian et al. [2011])**

The degenerate waveform consists of a "ping" which has three peaks and two troughs followed by a space of zero volts. Initially the ping appears to be characteristic of damped harmonic motion, with a damping ratio of 0.13 ± 0.02, but the attenuation then increases substantially, and the third peak is much lower than expected from a linearly damped oscillator. Overall the damping factor is 0.22 ± 0.09.

The signal was obtained from a registered electrical stimulatory device called the Fenzian. Thus device is currently in clinical use. The Fenzian signal from the device was digitized several times using a TDS 3012B digital phosphor oscilloscope from Tektronix (UK). The measured signals were saved and transferred to a standard PC. An in-house programme written using National Instruments' LabVIEW programming environment (National Instruments, Austin TX, USA) enabled us to read the data, allowing selection of a typical example with minimal noise. The ping was cut from the data, and inserted into a blank waveform, so ensuring that during the space the output is exactly zero. The programme was then transferred. This cleaned result was transferred as a custom waveform to an Agilent 33220A arbitrary waveform generator, which was used to generate the wave signal at a range of amplitudes and frequencies required for the experiment.

**Electric field calculation for degenerate wave stimulation in the *in vitro* electrical stimulation model**

Desired field strength was determined by dividing the voltage obtained on the oscilloscope by 25 mm (since 25 mm was the distance between the electrodes). For achieving different fields, for eg., 75 mV/mm field strength inside the chamber, approximately 2.6 mA of average current was supplied for Fig 1A, which resulted in 1.875 V across the chamber (between the electrodes) and 60 V delivered across the power supply.

**Supplementary Data S2**

Polystyrene petri dishes (35 mm x 10 mm) were used for subjecting cell-grown slides to capacitive coupling (CC) stimulation. The required numbers of cell-grown slides (~12) were placed in the petri dish before any attachment of the chamber to electrical equipment. The capacitors were connected according to figure 1C and the remaining electrical connections were set afterwards. Capacitors consisted of a pair of high-grade steel electrodes, each having a diameter of 8 cm, mounted in a plastic insulating material. The petri dish (with the cell seeded slides) was placed on the lower electrode and the upper electrode was placed above the medium leaving an air gap of ~2mm. This comprises the total separation between the electrodes to ~7.7.mm. The whole system was placed in a 5% CO_2_ controlled and 37°C temperature-regulated incubator box as explained for DW. To deliver CC, degenerate waves were generated from the frequency generator, which had a pulse width of 62.5 ms and a frequency of 16 Hz. This signal was amplified and fed to the capacitors. The voltage was adjusted on the frequency generator in such a way that the capacitors received 160 mV.

The electric field calculation between the capacitors was done using FEMLAB 3.5 (Comsol Multiphysics Inc.). Femlab simulation was used to understand approximate magnitude of electric field on a single cell. Polystyrene, glass, cell growth medium, cell membrane, steel and air were given relative permittivities as subdomain parameters with values of 2.6, 5, 77, 9, 3 and 1 respectively. Adequate thickness was assigned to each domain as used in the actual experiment (Supplementary Fig B in Supplementary Fig S1). In boundary settings, two identical materials were assigned “continuity” and other materials were assigned definite electric potential (160 mV for capacitors) and ground charge. Normal meshing parameters (software’s default) was applied to generate a mesh. The generated mesh was further refined. The model was resolved using the default stationary linear solver. After solving the problem, a smooth surface plot of the electric potential was displayed and was further customised with the help of “plot parameters” in the post-processing option. Voltage peaks of 103 µV were observed across the cell monolayer when 160 mV was applied to the capacitors, which gave a desired resultant electric field of 10 mV/mm across the cell which was observed with Femlab electric field simulation (Supplementary Figs A-D in Supplementary Fig S1). So, the electric field experienced by a single cell was determined as (~103 µV / 10 µm), which resulted in 10 mV/mm across the cell. 10 µm was the average thickness of a cell in the simulation studies. The actual electric field across the cell depends on multiple factors such as cell orientation/alignment to the field lines, neighbouring cells, particular cell cycle, physical characteristics etc. Therefore the electric field can change minimally between different cells. This could be one of the limitations of electric field simulation studies.

**Supplementary References**

Sebastian A, Farhatullah S, Mc Grouther DA, Colthurst J, Paus R, Bayat A. 2011. A novel in vitro assay for electrophysiological research on human skin fibroblasts: Degenerate electrical waves down regulate collagen I expression in keloid fibroblasts. Exp Dermatol.20:64-68.
