# Supplementary figures and images for: Expression of Concern: Enhancement of Differentiation and Mineralisation of Osteoblast-like Cells by Degenerate Electrical Waveform in an In Vitro Electrical Stimulation Model Compared to Capacitive Coupling
Source: PLoS One. 2026 Jan 20;21(1):e0341207. doi: 10.1371/journal.pone.0341207 (PMC12818591; doi:10.1371/journal.pone.0341207)

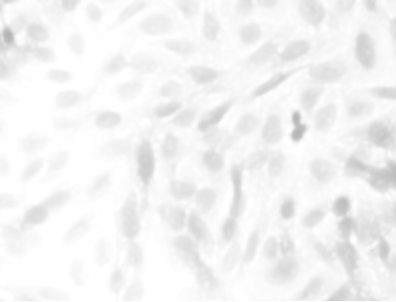

Supplement: S2 File — (ZIP) [file pone.0341207.s002.zip › DW28hr.4.tiff]

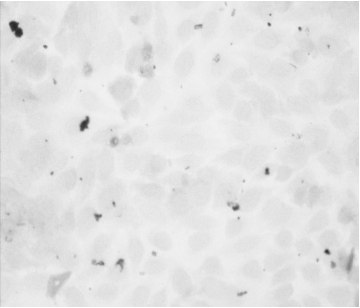

Supplement: S2 File — (ZIP) [file pone.0341207.s002.zip › CC28hr.1.tiff]

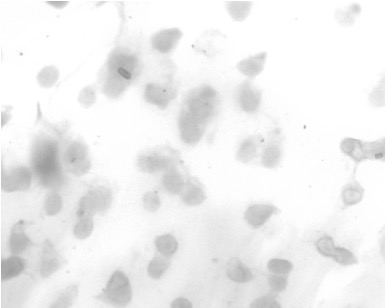

Supplement: S2 File — (ZIP) [file pone.0341207.s002.zip › CC28hr.2.tiff]

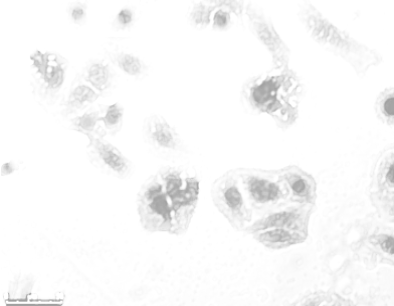

Supplement: S2 File — (ZIP) [file pone.0341207.s002.zip › CC28hr.3.tiff]

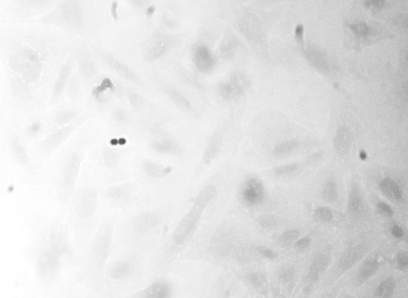

Supplement: S2 File — (ZIP) [file pone.0341207.s002.zip › CC28hr.4.tiff]

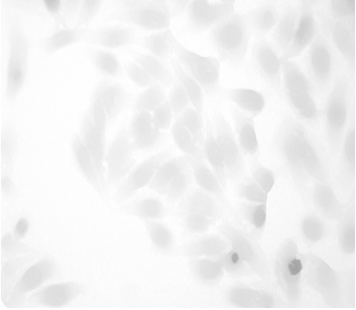

Supplement: S2 File — (ZIP) [file pone.0341207.s002.zip › control0hr.1.tiff]

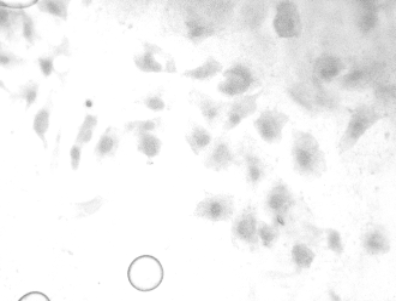

Supplement: S2 File — (ZIP) [file pone.0341207.s002.zip › control0hr.2.tiff]

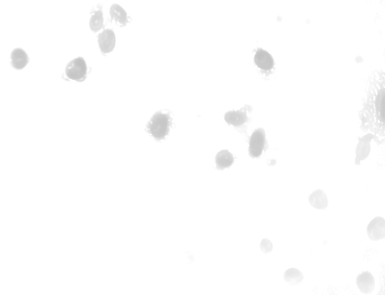

Supplement: S2 File — (ZIP) [file pone.0341207.s002.zip › control0hr.3.tiff]

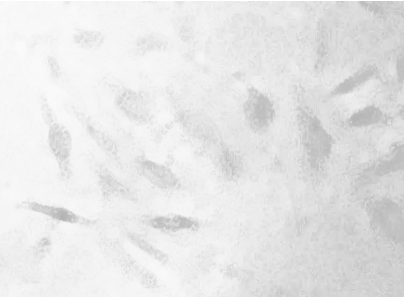

Supplement: S2 File — (ZIP) [file pone.0341207.s002.zip › control0hr.4.tiff]

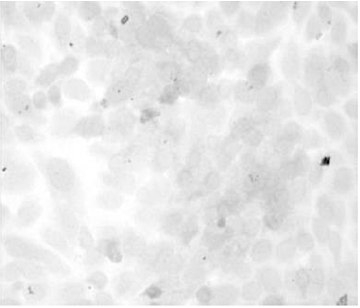

Supplement: S2 File — (ZIP) [file pone.0341207.s002.zip › control28hr.1.tiff]

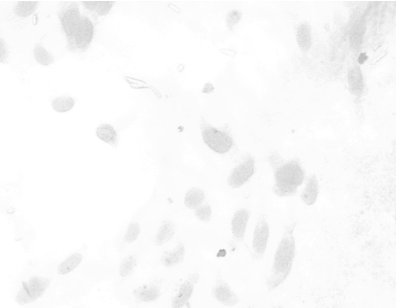

Supplement: S2 File — (ZIP) [file pone.0341207.s002.zip › control28hr.2.tiff]

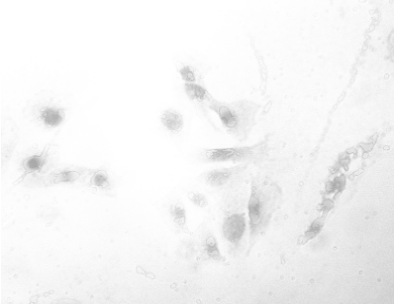

Supplement: S2 File — (ZIP) [file pone.0341207.s002.zip › control28hr.3.tiff]

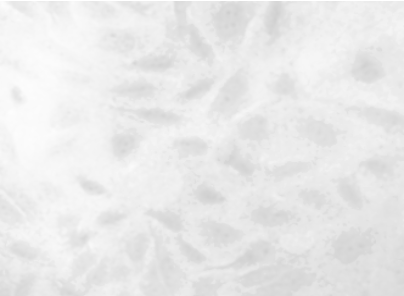

Supplement: S2 File — (ZIP) [file pone.0341207.s002.zip › control28hr.4.tiff]

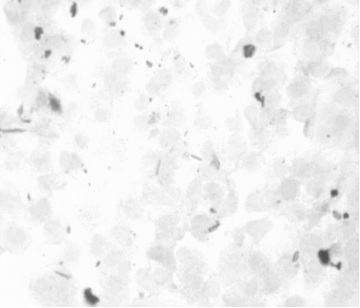

Supplement: S2 File — (ZIP) [file pone.0341207.s002.zip › DW28hr.1.tiff]

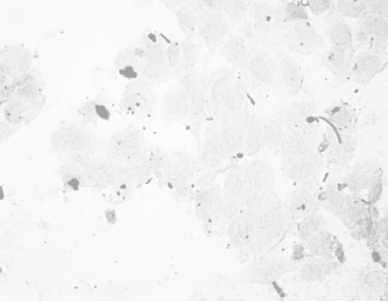

Supplement: S2 File — (ZIP) [file pone.0341207.s002.zip › DW28hr.2.tiff]

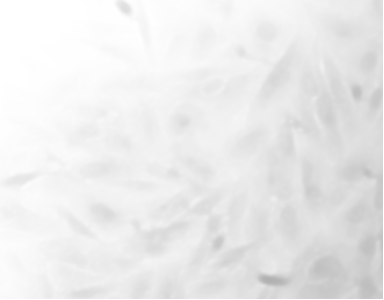

Supplement: S2 File — (ZIP) [file pone.0341207.s002.zip › DW28hr.3.tiff]
